# Serum DIP2C protein concentration in human colorectal cancer among Yemeni patients

**DOI:** 10.3389/fonc.2026.1918081

**Published:** 2026-08-20

**Authors:** Gamal Othman Dabwan, Zaid Hezam Al-Hamodi, Afif Al-Nabhi

**Affiliations:** 1 Department of Biochemistry and Molecular Biology, Faculty of Medicine and Health Sciences, Sana’a University, Sana’a, Yemen; 2 Department of Biochemistry and Molecular Biology, Faculty of Medicine and Health Sciences, Sana’a University, Sana’a, Yemen; 3 Department of Medicine, Faculty of Medicine and Health Sciences, Sana’a University, Sana’a, Yemen

**Keywords:** alternative splicing, CRC, DIP2C, khat, lifestyles, smoking, transcription, translation

## Abstract

**Background:**

Colorectal cancer (CRC) forms a serious health challenge in developed countries. In Yemen, khat (*Catha edulis*) chewing and smoking represent a common health risk, raising the risk of insulin resistance and potentially stimulating malignant tumors. However, studies of the *DIP2* family, especially *DIP2C*, are virtually absent in the Middle East, notably in Yemen, so this study focused on serum *DIP2C* levels in the context of colorectal cancer and khat chewing and smoking habits.

**Objective:**

This study aimed to investigate the serum *DIP2C* protein levels in CRC patients and to evaluate the influence of malignant patterns, age, BMI, smoking, and khat chewing on its concentration.

**Methods:**

In this study, 214 Yemeni adults aged 20–90 years were enrolled, including 73 CRC patients (25 with MetS and 48 without MetS) and 141 non-cancerous individuals (70 non-CRC, non-MetS subjects served as controls, and 71 non-CRC, MetS subjects). *DIP2C* levels were evaluated in relation to tumor risk factors, including age, body mass index (BMI), smoking, and khat use. Diagnostic performance was evaluated using ROC analysis.

**Results:**

Serum *DIP2C* levels were significantly lower in CRC patients with and without MetS compared to control individuals (*P = 1.5×10–^7^* and *0.043*). No significant differences in *DIP2C* levels were observed across subgroups stratified by MetS status, age, BMI, smoking status, or khat chewing status among non-CRC participants. ROC analysis demonstrated moderate discriminative performance, with an AUC of 0.755 (95% CI: 0.686–0.823), a sensitivity of 80.8%, and a specificity of 60.3%.

**Conclusion:**

Yemeni patients with colorectal cancer have noticeably low serum *DIP2C* protein levels with a sensitivity of 80.8%, a specificity of 60.3%, and a cut-off of 0.425. However, further functional studies are necessary to fully understand the potential of serum Dip2C as a biomarker for colorectal cancer (CRC) and its use as a diagnostic test for CRC.

## 1 Introduction

Colorectal cancer (CRC) poses a severe challenge to human health (1). It is the third most common cancer in humans (2–4), the second leading cause of cancer death worldwide (5, 6), and claims over half a million lives annually (7). The highest incidence rates of CRC are found in developed countries (8).

Khat consumption (9), water pipe smoking, and other forms of smoking are prevalent in the Arabian Peninsula, particularly in Yemen (10), increase oxidative stress (11), and raise concerns about their role in cancer (12, 13).

CRC is a heterogeneous disease that arises from the accumulation of genetic and epigenetic mutations that transform normal glandular epithelial cells into benign neoplasms (adenomas). Chromosomal instability (CIN), microsatellite instability, aberrant DNA methylation, and DNA repair defects are genomic instabilities that contribute to malignant transformation (14). Additionally, this tumorigenesis may be connected to metabolic disorders including type-2 diabetes (T2D), which are marked by elevated glucose levels and insulin resistance (IR) (15). The *DIP2C gene* has epigenetic signatures of CRC in visceral adipose tissue (VAT), and expression of *the* Disco-interacting protein 2 C gene (*DIP2C gene*) was related to the development of carcinogenesis (16). However, studies on the *DIP2* family, particularly *DIP2C*, remain scarce (17) and are largely limited to regions such as China and are nonexistent in the Middle East, particularly Yemen. This is especially true for research on *DIP2C* in the context of colorectal cancer, obesity, aging, and Yemeni social behaviors such as khat chewing. This is particularly relevant because khat chewers often consume tobacco heavily during khat chewing, which may amplify the effect.

DIP2C belongs to the DIP2 family in mammals, which includes three paralogs: DIP2A, DIP2B, and DIP2C (18). A single DIP2 ortholog exists in invertebrates such as Drosophila melanogaster (19) and Caenorhabditis elegans (20). In mammals, the three DIP2 proteins (DIP2A, DIP2B, and DIP2C) (18, 21) are encoded by three distinct genes (22), called DIPA, DIP2B, and DIP2C (17, 23). In humans, the DIP2C gene is located at 10p15.3, while the DIP2A and DIP2B genes are located at 12q13.12 and 21q22.3, respectively. These proteins possess one DMAP1-binding domain and two AMP-binding domains, conserved functional domains associated with acetyl-CoA synthesis (24, 25). DIP2C is a 1556-amino-acid polypeptide (26, 27) containing domains that bind the transcriptional corepressor DNA methyltransferase 1-associated protein 1 (DMAP1) (28). This protein is involved in lipid metabolism (29), especially fatty acid metabolism (30), and in obesity and cancer (16). DIP2C is essential for heart development, particularly cardiac looping (31), and mutations in DIP2C are linked to heart malformations, developmental delays with reduced expressive language, and seizures (19).

## 2 Materials and methods

### 2.1 Study design and participants

This case–control study included 214 Yemeni adults aged 20–90 years enrolled between 2023 and 2024 from the endoscopy units at Sana’a city, Yemen. Participants were divided into four groups: healthy controls (n = 70), the metabolic syndrome (MetS) group (n = 71), and colorectal cancer patients (25 with MetS and 48 without MetS).

MetS was defined according to the American Association of Clinical Endocrinology criteria (32–34) as IGT or IFG plus any of the following based on clinical judgment: TG ≥ 150 mg/dL and HDL-C < 40 mg/dL (men) or <50 mg/dL (women), BMI ≥ 25 kg/m2, SBP ≥ 130, DBP ≥ 85 mmHg, or treatment. The CRC diagnosis was confirmed by endoscopy and a histopathology test. Individuals without clinical features of MetS or CRC were selected as healthy controls after undergoing endoscopy and histological analysis to ensure no findings of CRC, benign tumors, or inflammatory lesions.

Subgroup analyses were performed according to smoking status, Khat chewing habit, BMI was categorized (35) as normal weight (18.5-24.9 kg/m², control group), underweight (< 18.5 kg/m^2^), overweight (25.0-29.9 kg/m²), or obese (≥30.0 kg/m²), and age group (< 40, 40–49, ≥ 50 years). Demographic and lifestyle data, including age, sex, smoking, and Khat chewing, were collected using a structured questionnaire. Height and weight were measured, and BMI was calculated as weight/height².

### 2.2 Inclusion and exclusion criteria

This study included CRC patients, non-CRC with MetS, non-CRC smokers, a non-CRC khat chewing group, and a control group of non-MetS and non-cancerous people. Cases that potentially impact the excretion and metabolism of serum *DIP2C* were eliminated from all study groups, including renal failure, abnormal liver functions (e.g., liver cirrhosis), heart conditions, protein-losing enteropathies, polyps, IBD, and chemotherapy or radiation therapy. In the CRC group, no blood samples were taken from those with cancers other than colorectal cancer. The control group did not include the hemorrhoids, intestinal tuberculosis, DM, or Mets participants or inflammatory lesions.

### 2.3 Ethical approval

The Medical Ethics Committee of the Faculty of Medicine and Health Sciences, Sana’a University (26; 13-7), approval date: 8/5/2023, approved the study. Written informed consent was obtained from all participants before enrollment.

### 2.4 Sample collection and storage

Venous blood samples (5 mL) were collected from participants. Serum was separated within 30 min, aliquoted, and stored at −80 °C until analysis. Samples were obtained from CRC patients, non-cancerous MetS, and healthy individuals undergoing screening colonoscopy.

### 2.5 Measurement of serum DIP2C levels

Serum DIP2C concentrations were measured using a commercial Human DIP2C ELISA kit (Cat. No. E7885Hu, BT LAB, China) following the manufacturer’s protocol. Standards ranging from 0.5 to 32 ng/mL were prepared, and the standard diluent served as the zero standard. All samples were assayed, and absorbance was measured at 450 nm with a HumanReader HS microplate reader (Human, Germany). Each serum sample was assayed in duplicate using the same reagent batch, with laboratory personnel blinded to patient status. Patient samples and controls were measured in the same ELISA run, and when more than one run was needed, controls were randomly distributed across plates. A point-to-point curve was automatically generated by the ELISA reader for quantification, and the mean value was used for analysis. The assay detection range was up to 32 ng/mL. According to the manufacturer’s protocol, intra-assay CV is < 8% and inter-assay CV is < 10%.

### 2.6 Statistical analysis

BM SPSS Statistics version 23 was used for statistical analysis, while figures were designed by GraphPad Prism version 10. The Kolmogorov-Smirnov test was utilized to determine normality. Serum *DIP2C* concentration data were not normally distributed. Values were transformed using a rank-based inverse-normal transformation before parametric analyses. Data are presented as geometric means with 95% confidence intervals. Group comparisons for *serum DIP2C* levels were made using univariate general linear models (GLM).

In analyses comparing the four main groups (Control, MetS, CRC without MetS, CRC with MetS), serum DIP2C concentration was the dependent variable, and group was the fixed factor; age and sex were covariates. In subgroup analyses (e.g., by BMI category, smoking status), similar GLM models were used, with relevant covariates (for BMI analysis, age/sex; for lifestyle factors, age/sex/BMI, as appropriate). ANOVA assessed group comparisons of participants’ age, and pairwise group differences were evaluated with Sidak-adjusted *post hoc* tests when the overall model was significant. G*Power version 3.1.9.7 was used to conduct a *post hoc* power analysis for an ANCOVA with four groups and two variables (age and sex). The power was >99% (Power ≈ 1.00), based on the observed effect size of Cohen’s f = 0.517, computed from partial η² = 0.211 in the primary analysis, an alpha of 0.05, and total N = 214. ROC analysis was performed to determine AUC, sensitivity, specificity, and optimal cut-off value for distinguishing CRC from controls for assessment of the diagnostic utility of serum DIP2C. A two-sided P value < 0.05 was considered statistically significant for all tests.

## 3 Results

### 3.1 CRC patients are the oldest among the study groups

**Figure 1:**
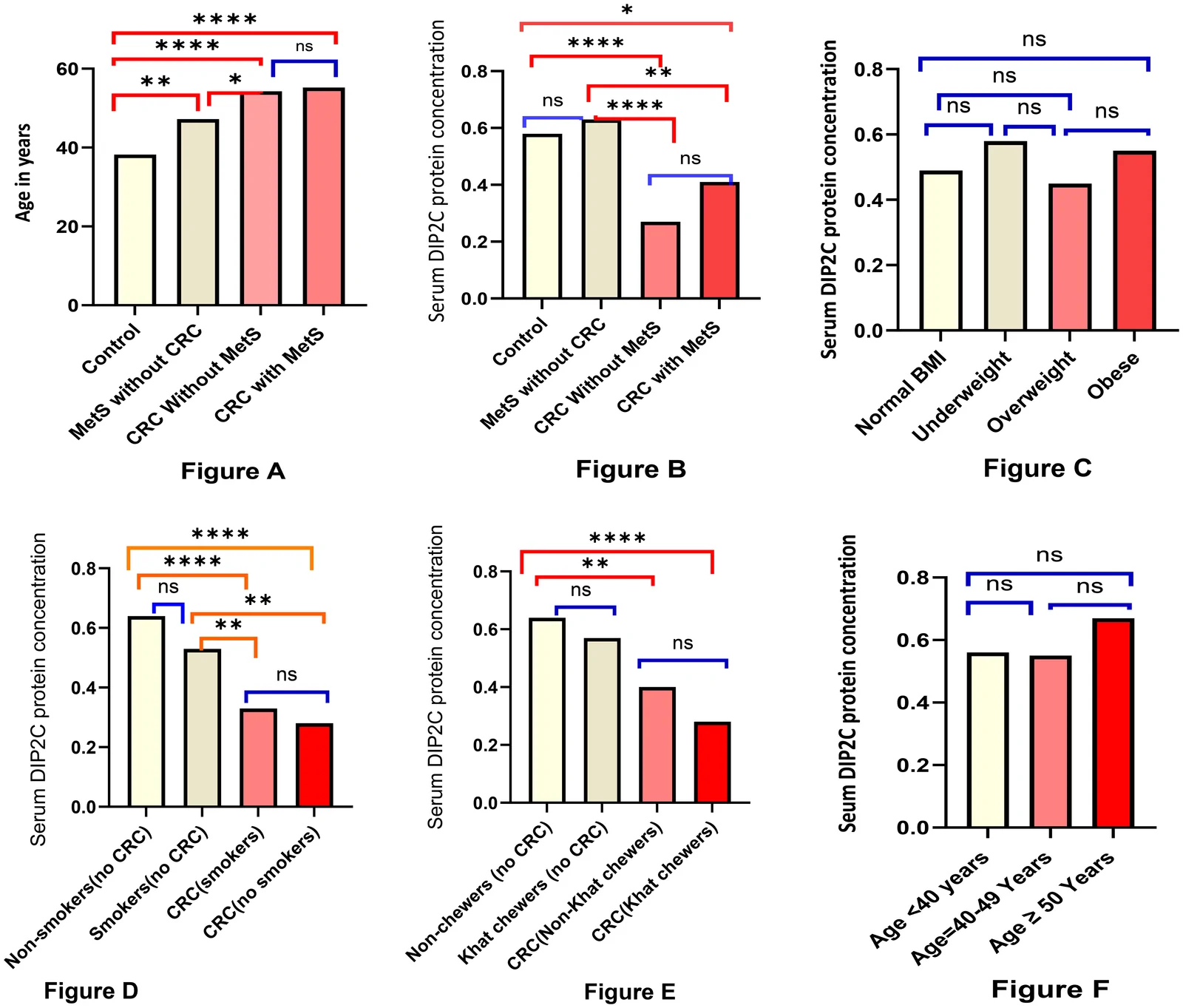
Represents serum DIP2C concentration across study groups and risk factors. **(A)** Age distribution of the main study groups. **(B)** Serum DIP2C concentration among the main study groups. **(C)** Serum DIP2C concentration across BMI categories. **(D)** Serum DIP2C concentration stratified by smoking status. **(E)** Serum DIP2C concentration stratified by Khat chewing status. **(F)** Serum DIP2C concentration across age groups. Data are presented as geometric means (95% confidence interval of mean) as appropriate for each analysis. Statistical significance is indicated as *P < 0.05, **P < 0.01, and ****P < 0.0001. CRC, colorectal cancer; MetS, metabolic syndrome; BMI, body mass index; DIP2C, disco-interacting protein 2 homolog C.

The CRC groups and non-CRC MetS were significantly older than the control group (**Table 1**; **Figure 1A**; *P < 0.01*), with the CRC group being the oldest (**Table 1**; *P < 0.00001*). Accordingly, age and sex were included as covariates in all subsequent analyses. Among CRC patients, 25 of 73 (34%) met the criteria for concomitant metabolic syndrome (**Table 1**).

**Table 1:** Summarizes age and clinical features.

Study group	n	Age in(years)	*P* vs. control	*P* vs. MetS without CRC group	*P* vs. CRC without MetS group	Age (years)		
						Age <40 years	Age (40-49) years	Age ≥50 years
Control	70	38.2 (34.4- 42.0)	–	–	–	44 (62.9%)	8 (11.4%)	18 (25.7%)
MetS without CRC	71	47.2 (43.8- 50.5)	**0.003**	–	–	27 (38%)	18 (25.4%)	26 (36.6%)
CRC without MetS	48	54.3 (50.5- 58.7)	**9.0 ×10^-8^**	**0.047**	-	8 (16.7%)	8 (16.7%)	32 (66.7%)
CRC with MetS	25	55.2 (49.3- 61.1)	**1.0 × 10^-6^**	0.12 (ns)	0.99 (ns)	5 (20%)	2 (8%)	18 (72%)
CRC (total)	73	54.8 (51.5-58.1)	**5.6× 10^-10^**	**0.007**	–	13 (17.8%)	10 (13.7%)	50 (68.5%)

Bold values represent statistical significance (p < 0.05).

68.5% of the CRC patients were ≥50. The majority of individuals in the control group (62.9%) were under 40 years old, whereas 60% of MetS patients were ≥ 40 years old. Younger people (less than 40 years old) made up 17.8% of CRC patients.

### 3.2 Serum DIP2C levels are reduced in colorectal cancer, independent of metabolic syndrome

Serum *DIP2C* was significantly lower in CRC patients compared to controls with normal colorectal mucosa and non-cancer individuals with MetS criteria (**Table 2**).

**Table 2:** *DIP2C* concentration by main study group (values are age- and sex-adjusted).

Study group	n	Serum *DIP2C* concentration (ng/ml)	*P* vs. control	*P* vs. MetS without CRC group	*P* vs. CRC without MetS group
Control	70	0.58 (0.52- 0.65)	–	–	–
MetS (without CRC)	71	0.63 (0.57- 0.69)	0.86 (ns)	–	–
CRC (without MetS)	48	0.27 (0.19- 0.35)	***1.5×10^-7^***	***1.6×10^-10^***	*-*
CRC with MetS	25	0.41 (0.30- 0.51)	***0.043***	***0.002***	0.22 (ns)
CRC group (total)	73	0.32 (0.25-0.38)	***5.4×10^-7^***	***3.1×10^-10^***	–

Ns, not significant, p value = *1.1×10^-10.^*

Bold values represent statistical significance (p < 0.05).

Levels were reduced by approximately 50–70% in CRC patients both with MetS (P = *0.043*) and without MetS (P = *1.5×10^-7^*) relative to controls, and these percentages were also similar in CRC patients both with MetS (P = *0.002*) and without MetS (P = *1.6×10^-10^*) compared to non-cancer individuals with MetS. In contrast, *DIP2C* concentrations did not differ between controls and non-CRC MetS subjects, nor between CRC patients with and without MetS (**Table 2**). These data indicate that reduced serum *DIP2C* is associated with colorectal adenocarcinoma rather than metabolic syndrome alone (**Figure 1B**). Despite CRC with MetS having a small sample size, however, serum DIP2C in CRC patients(total) was significantly lower. The mean of the CRC group (total) was 0.32 (0.25-0.38) vs 0.58 (0.52-0.65) in controls, p = *5.4×10⁻^7^.*

### 3.3 Serum *DIP2C* levels are not linked with BMI

Analysis across WHO BMI categories showed no significant difference in serum *DIP2C* levels after adjusting for age, sex, fasting glucose, lifestyle factors, and cancer status (overall P = 0.098, **Table 3**).

**Table 3:** Serum *DIP2C* concentration across BMI categories (ns).

BMI category	Definition (kg/m^2^)	n	Serum *DIP2C* protein (geometric mean, 95% CI)	*P* vs. normal BMI	*P* vs. underweight	*P* vs. overweight
Normal	18.5-24.9	89	0.49 (0.44- 0.55)	–	–	–
Underweight	< 18.5	27	0.58 (0.48- 0.68)	0.53 (ns)	–	–
Overweight	25.0-29.9	50	0.45 (0.38- 0.52)	0.92 (ns)	0.19 (ns)	–
Obese	≥ 30.0	48	0.55 (0.47- 0.63)	0.79 (ns)	0.99 (ns)	0.29 (ns)

ns, not significant. The overall P value was 0.098.

Thus, serum *DIP2C* concentration correlated with CRC status but not with BMI (**Figure 1C**).

### 3.4 Smoking and khat chewing do not alter serum *DIP2C* levels in individuals without CRC

Serum *DIP2C* concentrations did not differ between smokers and non-smokers, or between khat chewers and non-chewers, in participants with normal colorectal mucosa (**Tables 4**, **5**; **Figures 1D, E**), which indicates neither tobacco nor khat chewing changes serum *DIP2C* concentration in individuals with normal colorectal mucosa.

**Table 4:** Serum *DIP2C* protein concentration through smoking status. (ns = not significant).

Group	n	*DIP2C* (geometric mean, 95% CI)	*P* vs. reference group	*P* vs. smokers (non-CRC)	*P* vs. CRC (non-smokers)
Non-smokers (no CRC) reference group	104	0.64 (0.58- 0.69)	–	–	–
Smokers (non-CRC)	37	0.53 (0.44- 0.68)	0.24 (ns)	–	–
CRC (non-smokers)	51	0.33 (0.25- 0.40)	***4.4×10^-9^***	***0.005***	–
CRC (smokers)	22	0.28 (0.16- 0.40)	***0.4×10^-5^***	***0.009***	0.99 (ns)

Ns, not significant. Overall P value was *7.6×10^-10.^*

Bold values represent statistical significance (p < 0.05).

**Table 5:** Serum Dip2c protein concentration through Khat chewing status.

Group	n	*DIP2C* protein (geometric mean, 95% CI)	*P* vs. control (no chewers)	*P* vs. Khat chewers (no CRC)	*P* vs. CRC (non- chewers)
No-chewers (control)	67	0.64 (0.58- 0.71)	–	–	–
Khat chewers (no CRC)	74	0.57 (0.51- 0.64)	0.58 (ns)	–	–
CRC (non- chewers)	23	0.40 (0.28- 0.51)	***0.002***	0.063	–
CRC (chewers)	50	0.28 (0.20- 0.36)	***4.7×10^-10^***	***9.3×10^-7^***	0.41

Ns, not significant. overall p value = 5.6×10^-10.^

Bold values represent statistical significance (p < 0.05).

In contrast, *DIP2C* levels were significantly lower in CRC patients regardless of smoking or khat chewing status compared to non-cancer controls (P < *10⁻^4^* for smoking; P < *0.005* for khat chewing).

### 3.5 Serum *DIP2C* levels are not associated with age (ns)

No significant difference in serum *DIP2C* concentration was observed across age groups <40, 40–49, and ≥50 years in individuals with normal colorectal mucosa (overall P = 0.105, **Table 6**; **Figure 1F**).

**Table 6:** Serum *DIP2C* protein concentration through age groups.

Age group	n	*DIP2C* concentration (geometric mean, 95% CI)	*P* vs. < 40 yrs
< 40 years	71	0.56 (0.50- 0.63) (reference)	–
40–49 years	26	0.55 (0.44- 0.65)	0.99 (ns)
≥ 50 years	44	0.67 (0.58- 0.75)	0.17 (ns)

Ns, not significant.

### 3.6 Serum DIP2C’s diagnostic performance in differentiating CRC patients from healthy individuals

ROC curve analysis was used to assess DIP2C’s diagnostic performance for CRC. In this single study from Yemen, serum *DIP2C* showed moderate diagnostic accuracy, with an AUC of 0.755 (95% CI: 0.686–0.823; *p < 0.00001*) (**Table 7**; **Figure 3**). At the optimal cut-off of *0.425, DIP2C* distinguished CRC from normal tissues with a sensitivity of 80.8% and a specificity of 60.3%, which requires validation in independent populations.

## 4 Discussion

In the present study, the majority of CRC patients were in the oldest age. Previous research indicates that those over 50 account for 90% of colorectal cancer occurrences, and the disease’s incidence rises with age (36), which is linked to changes in bile acid formation and composition with colorectal tumorigenesis (37).

**Figure 2:**
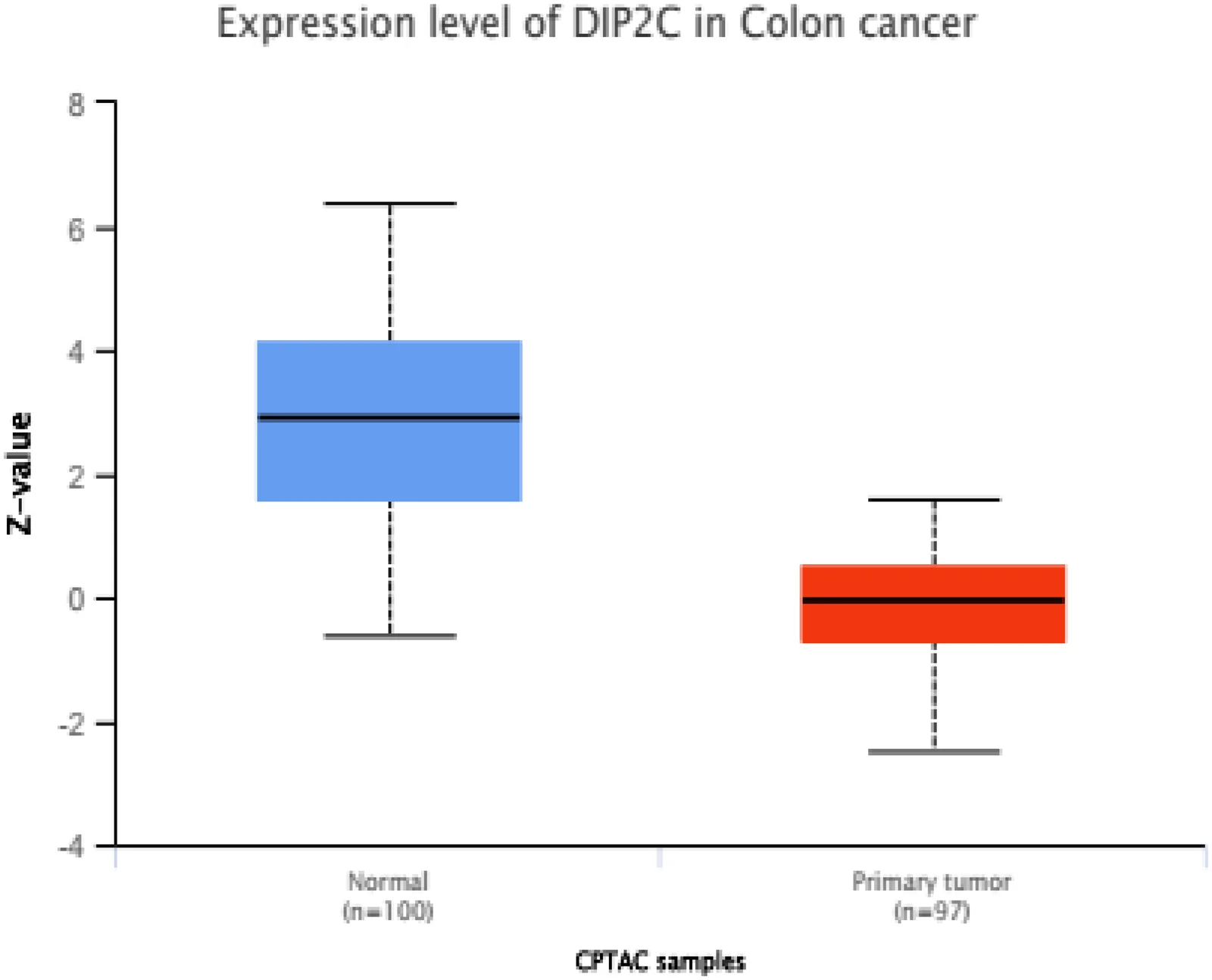
Represent CPTAC proteomic data of tissue DIP2C in CRC.

The protein levels balance lies between synthesis and clearance. Synthesis rates depend on mRNA abundance and translational efficiency, whereas clearance involves degradation, secretion, and its consumption due to cell growth (38). Post-transcriptional regulation, including mRNA splicing, transport, and stability, may influence protein synthesis (39, 40). In this study, serum *DIP2C* concentrations were considerably lower in colorectal cancer patients than in healthy controls, representing the first description of *DIP2C* levels in a Middle Eastern, Yemeni sample. Serum DIP2C levels in CRC patients ranged from 50% to 70% of those in healthy individuals. This suggests that the downregulation observed in CRC is a tumor-specific variable rather than driven by lifestyle factors alone. The downregulation of *DIP2C* observed in CRC patients in the current study is consistent with the DNA hypomethylation pattern previously reported in CRC and corresponds with TCGA (The Cancer Genome Atlas) data reports of DNA hypomethylation in Taiwanese and Western CRC populations (41). The substantial downregulation of serum *DIP2C* in CRC patients is the study’s most significant discovery. DIP2C levels were not impacted by MetS alone (p = 0.86); however, this decrease was observed in CRC patients with MetS (p = 0.043), those without MetS (p = 1.5×10⁻7), and the entire CRC group (p = 5.4×10^-7^) in comparison to controls. This implies that colorectal cancer is specifically linked to decreased serum *DIP2C*. The CPTAC (Clinical Proteomic Tumor Analysis Consortium) proteomic data (**Figure 2**), which revealed significantly reduced DIP2C in malignant colorectal tissues (n = 97) compared to healthy controls (n = 100), support this serum finding. *DIP2C* depletion in cancer tissue may explain the lower serum levels, given the link between tissue and circulating protein levels. The fact that *DIP2C* levels did not correlate with BMI categories provides additional evidence that the change is related to cancer rather than obesity. One possible explanation for Increased protein breakdown is aberrant gene expression and protein folding defects (42). MicroRNAs and RNA-binding proteins may also play a role in post-transcriptional regulation. For example, HuR, an RNA- binding protein overexpressed in colon cancer tissues, promotes mRNA destabilization and translational repression (40). The lower serum DIP2C found in Yemeni CRC patients may be attributed to dysregulation of these processes.

The DMAP1-binding domain of *DIP2C* has an N-terminus that interacts with DNA methyltransferases (DNMTs) (43, 44) and performs DNA methylation within the cell (45). Given that promoter hypomethylation is a hallmark of colorectal cancer (46, 47). This epigenetic change may be mechanistically connected to the downregulation of DIP2C seen in this study’s CRC sample. The low blood DIP2C levels in the CRC cases in the current study are thought to be consistent with earlier findings of worldwide DNA hypomethylation in CRC. However, as the DNA methylation state of the DIP2C gene was not assessed in this cohort, this process remains speculative and requires further examination in future epigenetic studies.

Alternative splicing generates multiple mRNA isoforms and increases transcriptome complexity from a single gene (48–50). However, most alternative transcripts are not translated into stable proteins (51), leading to a frequent discordance between mRNA and protein abundance. This discordance is largely driven by post-transcriptional mechanisms, which account for approximately 60% of the variation in protein levels, compared to 40% attributable to mRNA abundance (52). Translation rates can also be heterogeneous, further decoupling protein output from transcript levels (53). Post-transcriptional mechanisms such as translational control and alternative splicing may hypothetically contribute to the decreased serum DIP2C levels observed in CRC patients. Hypothetically, low serum DIP2C could be explained through altered translation efficiency, mRNA instability, or abnormal protein secretion in tumor cells. However, no experimental data on these pathways were collected in this study, and further mechanistic validation is required.

Aberrant alternative splicing is a common characteristic of cancer (54), including CRC (55), and is relevant for both diagnosis and treatment (56). Alternative splicing was observed in malignancies (57) and in mutated *DIP2C* (58). Poor translation or unstable mRNA isoforms may arise from dysregulation of alternative splicing. Thus, it is believed that this mechanism may help explain the decrease in serum DIP2C observed in CRC patients in the current study. This proposed mechanism remains speculative and requires validation in future studies.

The development and progression of the malignant state depend on post-translational control in its various forms (59–61). The UPS regulates the stability of proteins, including those linked to cancer chemoresistance (62), through ubiquitination by E3 ligases (63) and deubiquitination by deubiquitinases (DUBs) (64). It is commonly known that dysregulation of this equilibrium occurs in colorectal cancer (CRC) and is associated with tumor formation and chemoresistance. Oncoprotein stability or aberrant tumor suppressor degradation can result from defective UPS gene regulation, which may contribute to malignant proliferation and the development of human cancers (65).

Based on these mechanisms mentioned in previous studies and not confirmed in the present study, the reduced serum DIP2C in CRC patients may be interpreted. These suggested and proposed mechanisms warrant further investigation. However, no experimental data on these pathways were collected in this study.

Khat chewing and tobacco use are prevalent in Yemen (66, 67) and associated with health disorders (68). They are associated with oxidative stress and persistent inflammation in many tissues (69–71), which can change the methylation of particular tumor suppressor genes in malignancies (72). Both habits can also alter protein synthesis and gene expression (73–76). Khat chewing reduces appetite and causes hyperthermia. Loss of appetite decreases body weight, BMI, and body mass (77), leading to damage of cellular proteins (78). This study hypothesized that exposure to these lifestyle factors would affect serum *DIP2C* levels. However, the findings do not show a direct influence of smoking or khat chewing on serum *DIP2C* levels. In non-cancerous individuals, *DIP2C* levels were comparable between smokers and non-smokers, as well as between khat chewers and non-chewers. Once CRC was established, serum *DIP2C* levels were persistently low regardless of smoking or khat use. Khat is associated with decreased appetite. Its cultivation is linked to the use of harmful chemicals and pesticides, which can cause cancer and gastrointestinal diseases; however, khat chewers must consume high-calorie foods to maintain satiety for an extended period of time (79).

In Yemen, Al-Fahsa and Saltah are regarded as the most popular traditional dishes. They are typically consumed with the noon diet and before chewing khat, and they are a major meal among Yemenis, particularly those who chew khat. Because of their high-calorie and protein content, these meals may compensate for lower food intake during chewing hours; they are an important part of this group’s nutritional evaluation. Chewing khat is often associated with reduced appetite throughout the day; thus, this one high-calorie meal could have a significant effect on the population’s overall nutritional health. The nutritional practices of Yemeni khat chewers may have shielded them from the undernutrition linked to the habit, which could explain why DIP2C levels remained unaffected. However, because precise food consumption and nutritional status were not systematically evaluated in this investigation, the suggested protective effect of specific high-calorie meals on nutritional status or DIP2C should be regarded as a hypothesis requiring specialized nutritional research.

These findings indicate that colorectal cancer is the primary pathway for *DIP2C* downregulation, and that lifestyle factors (smoking and khat chewing) do not directly influence serum *DIP2C* concentration in cancer-infected or non-cancer persons. When cancer develops (perhaps with the help of these variables), serum *DIP2C* protein content drops as a result of the carcinogenic process, not as a direct result of exposure. This distinction is clinically significant: it suggests that low serum *DIP2C* protein concentration may be a marker of malignant transformation rather than of exposure itself. But to validate these results and evaluate the temporal connection between DIP2C levels and CRC development, prospective, multi-center studies with complete anthropometric data are required.

**Figure 3:**
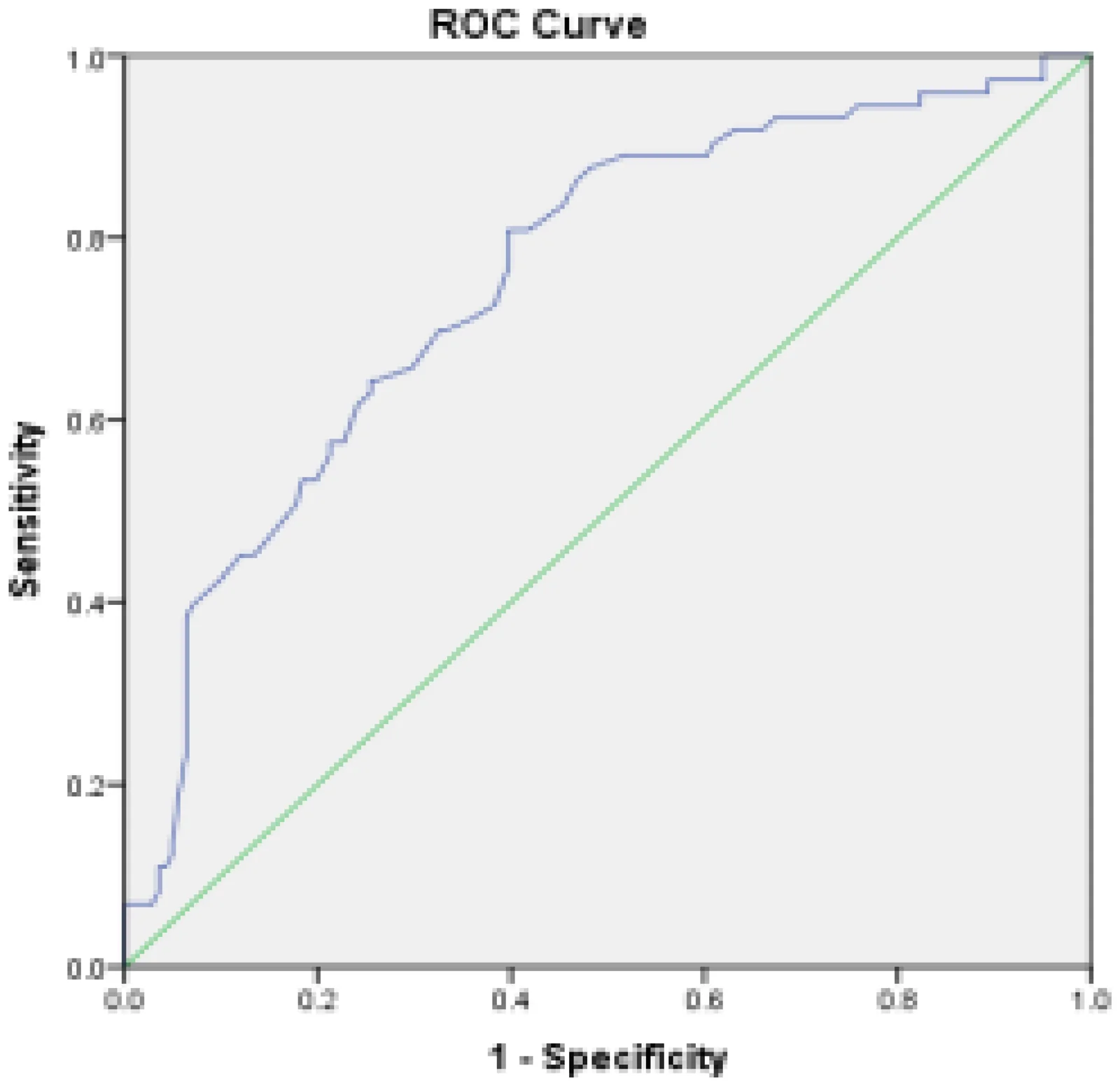
Represents an ROC curve of serum DIP2C in CRC.

Based on ROC analysis and serum CEA and CA19.9 diagnosis accuracy in colorectal cancer. CEA has an AUC of 0.623, a sensitivity of 40.0%, and a specificity of 85%, with a cut-off of 5 ng/mL. In contrast, CA19.9 has a cut-off of 37 U/mL, an AUC of 0.573, a sensitivity of 32.5%, and a specificity of 80.0% (80). Serum *DIP2C* has an AUC of 0.755, a sensitivity of 80.8%, a specificity of 60.3% with a cut-off of 0.425 ng/ml, and moderate diagnostic performance for CRC (**Table 7**; **Figure 3**). With a sensitivity of 80.8%, serum DIP2C may be used as a screening test to lower false negatives; nevertheless, a considerable percentage of false positives is indicated by its specificity of 60.3%. Therefore, when combined with well-known indicators like CEA (carcinoembryonic antigen), CA19.9, or FIT (fecal immunochemical test), serum DIP2C may be more helpful in a panel approach—future multi-center, large-scale validation. DIP2C had a moderate diagnostic accuracy for distinguishing CRC patients from controls despite ROC analysis. Nevertheless, before *DIP2C* can be regarded as a useful diagnostic, these ROC metrics, which were obtained from this particular Yemeni cohort, need to be validated in separate populations.

**Table 7:** Diagnostic performance of serum DIP2C.

AUC	95% CI	P value	Cut- off	Sensitivity	Specificity
0.755	0.686-0.823	**1 ×10^-9^**	0.425	80.8%	60.3%

AUC, Area Under the Curve.

### 4.1 Clinical benefit

The significant difference between CRC patients and healthy controls reveals that serum *DIP2C* has the potential to be a biomarker for CRC. However, validation entails additional efforts.

Tissue-serum correlation of *DIP2C* is confirmed by tissue immunohistochemistry or proteomics, which can determine whether low serum *DIP2C* levels associate with tumor events, intracellular retention, or defective secretion. Additionally, the *DIP2C* protein pathways should be studied through *DIP2C*’s post-transcriptional regulation, including ubiquitin-proteasome-mediated degradation and alternative splicing, which will provide insight into how carcinogenesis affects its levels. However, *DIP2C* levels as a biomarker for CRC are assessed by measuring serum *DIP2C* in premalignant lesions, such as adenomas, and by evaluating its levels across tumor stages, which may help identify it as a marker for early detection and determine whether alterations occur early. Also, this requires large, diversified cohorts to define normal values and evaluate their specificity independent of age, BMI, and lifestyle factors. Finally, longitudinal studies are required to determine whether *DIP2C* levels are connected to prognosis, treatment response, and disease progression. If validated, *DIP2C* may help with patient monitoring and risk identification. To measure *DIP2C*’s additional diagnostic value in a multi-marker panel, future prospective studies should assess this protein in screening or surveillance settings with well-known markers including CEA, CA19-9, and FIT.

## 5 Conclusion

**Figure 4:**
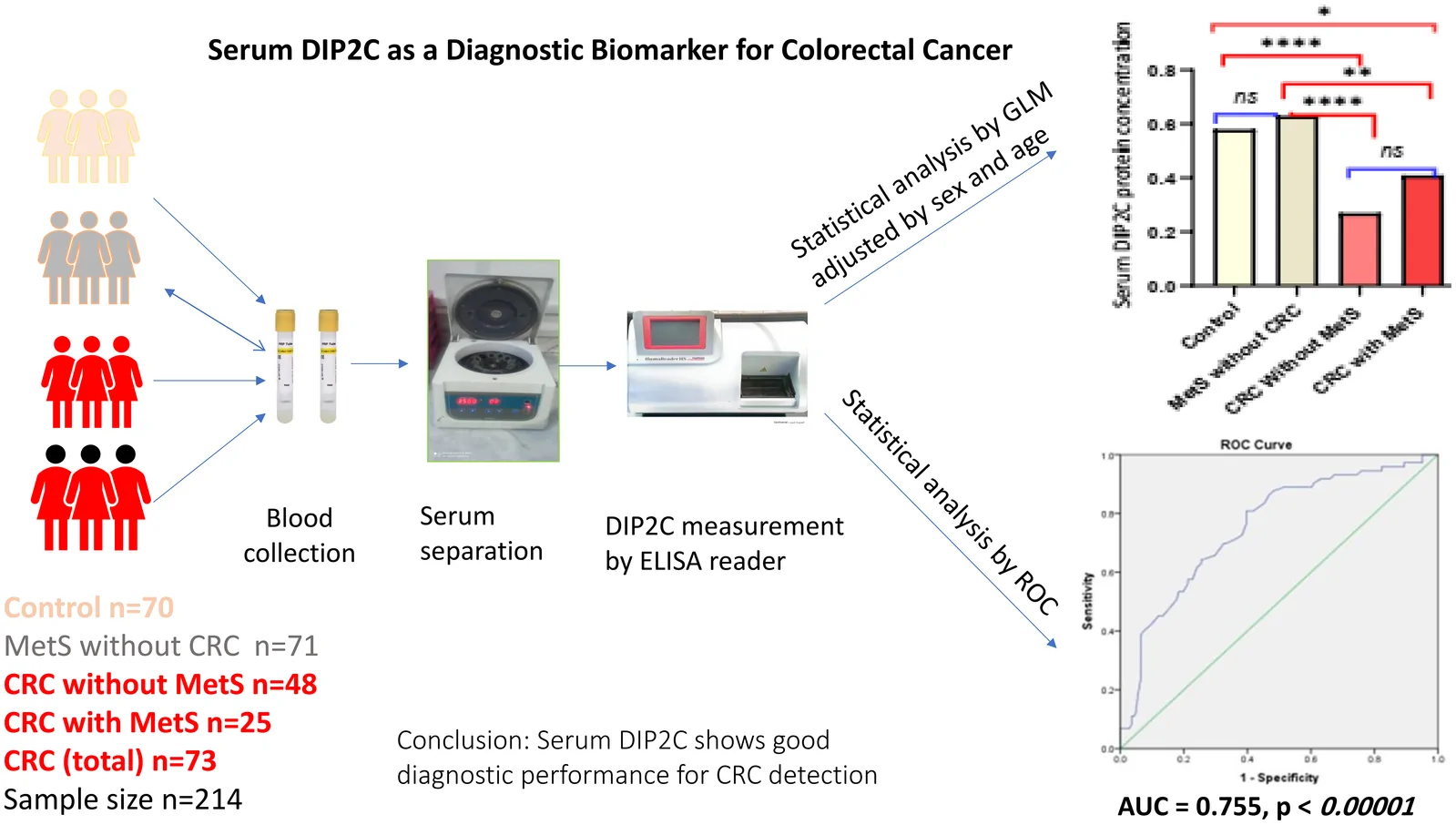
Graphical Abstract of the study design and main findings. A total of 214 participants were enrolled, including Control n=70, MetS without CRC (total) n=71, CRC without MetS n=48, and CRC with MetS n=25. Blood samples were collected, followed by serum separation and DIP2C measurement using ELISA. The bar graph shows serum DIP2C levels across groups with statistical analysis by GLM adjusted for sex and age. *P < 0.05, **P < 0.01, and ****P < 0.0001, ns = not significant. ROC curve analysis demonstrated good diagnostic performance of serum DIP2C for CRC detection with AUC = 0.755, p < 0.00001. Conclusion: Serum DIP2C shows moderate diagnostic performance for CRC detection.

This study shows that serum *DIP2C* is significantly downregulated in Yemeni CRC patients and is unrelated to age or common lifestyle exposures. It has moderate diagnostic performance for differentiating between CRC and healthy individuals. The Graphical Abstract (**Figure 4**) provides a summary of the study's design and key conclusions. According to this data, *DIP2C* could serve as a marker of malignant transformation and a target for early intervention. Although the exact molecular mechanisms underlying this decrease remain unknown, post-transcriptional and post-translational regulatory processes could be one explanation. Longitudinal studies should be performed to determine whether *DIP2C* levels are connected to prognosis, treatment response, and disease progression. If validated, *DIP2C* may help with patient monitoring and risk identification. Further studies in diverse populations and experimental models are needed before clinical application.

### 5.1 Strengths and limitations

Strengths of this study include the use of a well-characterized control group free of conditions known to alter serum protein levels, such as diabetes, metabolic syndrome, renal failure, abnormal liver function (liver cirrhosis), IBD, and protein-losing disorders. CRC diagnosis was confirmed by endoscopy and histopathology, and participants with polyps, other malignancies, or those receiving chemotherapy or radiotherapy were excluded. By using univariate general linear models (GLMs) to control for important confounding variables such as age, sex, obesity, smoking, and khat-eating behaviors, the study’s validity was enhanced.

The current study has several limitations. First, despite the power being> 99%, particular subgroups exhibited relatively small sample sizes, especially the CRC with MetS group (n=25). However, there were statistically significant variations in serum *DIP2C* between the groups. As a result, further research with larger, well-powered cohorts is advised to confirm and expand on these findings. Second, the single-center cohort and modest sample size in subgroup analyses may also limit generalizability to populations with different genetic backgrounds or exposure profiles. Prospective and multi-center studies with complete anthropometric data are needed to validate these findings and assess the temporal relationship between *DIP2C* levels and CRC development. Third, not every CRC patient had access to comprehensive clinicopathological characteristics, such as TNM stage, tumor grade, tumor location, and lymph node status. As a result, unable to ascertain whether the decrease in serum DIP2C levels is related to tumor burden or cancer stage. To solve this, more research with comprehensive staging data is required. Fourth, this study did not identify colorectal adenoma and inflammatory bowel disease as distinct categories, but they were excluded from the study. Therefore, it is still unknown how specific *DIP2C* is for cancer versus benign tumors and inflammatory diseases. Fifth, limitations include the cross-sectional design, which precludes causal inference between low serum *DIP2C* and colorectal carcinogenesis. Sixth, complete TNM staging and tumor grade were not available for all CRC patients. The current findings on *DIP2C* should be regarded as hypothesis-generating and require validation in staged cohorts before any conclusions regarding stage-specific behavior can be formed.

Finally, despite these limitations, findings of this study suggest that serum *DIP2C* may have potential as an adjunctive diagnostic marker for CRC and warrant validation in larger prospective studies.

## Data Availability

The raw data supporting the conclusions of this article will be made available by the authors, without undue reservation.
