# Correction: Serum DIP2C protein concentration in human colorectal cancer among Yemeni patients

**DOI:** 10.3389/fonc.2026.1982006

**Published:** 2026-09-28

**Authors:** Gamal Othman Dabwan, Zaid Hezam Al-Hamodi, Afif Al-Nabhi

**Affiliations:** 1 Department of Biochemistry and Molecular Biology, Faculty of Medicine and Health Sciences, Sana’a University, Sana’a, Yemen; 2 Department of Biochemistry and Molecular Biology, Faculty of Medicine and Health Sciences, Sana’a University, Sana’a, Yemen; 3 Department of Medicine, Faculty of Medicine and Health Sciences, Sana’a University, Sana’a, Yemen

**Keywords:** alternative splicing, CRC, DIP2C, khat, lifestyles, smoking, transcription, translation

Author “Zaid Hezam Al-Hamodi^2^, Afif Al-Nabhi^3^” was omitted as an author in the published article. The correct author list reads:

“Gamal Othman Dabwan^1^*, Zaid Hezam Al-Hamodi^2^, Afif Al-Nabhi^3^”

“^1^Department of Biochemistry and Molecular Biology, Faculty of Medicine and Health Sciences, Sana’a University, Sana’a, Yemen

^2^Department of Biochemistry and Molecular Biology, Faculty of Medicine and Health Sciences, Sana’a University, Sana’a, Yemen.

^3^Department of Medicine, Faculty of Medicine and Health Sciences, Sana’a University, Sana’a, Yemen.”

The **Author contributions statement** has been corrected to read:

“GD: Conceptualization, Methodology, Data curation, Formal analysis, Writing – original draft, Writing – review & editing, and Project administration.

ZHA: Supervision, Methodology, Formal analysis, Investigation, Resources, Data curation, Writing – original draft, Writing – review & editing, Project administration.

AN: Supervision, Writing – original draft, Writing – review & editing, Data curation, Validation.”

All authors approved the final version of the manuscript and agreed to be accountable for all aspects of the work.

The original version of this article has been updated.

